# 308. Injectable Scaffold-based Vaccination For Prevention of Periprosthetic Joint Infection

**DOI:** 10.1093/ofid/ofad500.380

**Published:** 2023-11-27

**Authors:** Alexander M Tatara, Shanda Lightbown, Hamza Ijaz, Michael Super, Sandra B Nelson, David Mooney

**Affiliations:** Massachusetts General Hospital, Boston, Massachusetts; Wyss Institute, Boston, Massachusetts; Wyss Institute, Boston, Massachusetts; Wyss Institute at Harvard University, Boston, MA; Massachusetts General Hospital, Boston, Massachusetts; Harvard University School of Engineering and Applied Sciences, Boston, Massachusetts

## Abstract

**Background:**

There are currently no vaccines available to prevent periprosthetic joint infection (PJI) and existing vaccine platforms have failed against *Staphylococcus aureus* in clinical trials. We have developed a novel injectable scaffold-based vaccine for PJI. In this study, we evaluated vaccine immune response and bacterial burden in a murine model of PJI.

**Methods:**

Vaccines consisting of mesoporous silica rods (scaffold) and demethylated CpG (adjuvant) were prepared using *S. aureus*-derived pathogen-associated molecular patterns (PAMPs) as described previously (Super and Doherty et al., *Nat Biomed Eng* 2022).

Mice were either unvaccinated or vaccinated by subcutaneous scaffold injection containing different amounts of PAMP antigen: 7.5, 30, or 150 units (n=5-8 per group). Mice received a second scaffold injection on Day 14 (same dose as Day 0) as booster. Sera was collected on Day 28. For a challenge study, mice vaccinated on Days 0 and 14 with scaffold loaded with 30 U of PAMPs (“Vaccinated”) underwent implantation of a stainless-steel wire in the distal femur protruding into the joint space on Day 35 (n=8). The implant was inoculated with 1000 CFU *S. aureus* Xen29, a bioluminescent strain. Infected mice without vaccination (“Unvaccinated”) and without infection (“Uninfected”) also underwent implant insertion (n=5). Mice underwent bioluminescent imaging, collection of sera and tissue, and euthanasia with implant harvest on Day 49.

**Results:**

Vaccination increased anti-*S. aureus* antibodies in a dose-dependent manner. Vaccination with Xen29 PAMPs resulted in antibodies with cross-reactivity against other *S. aureus* strains (RN4220, RN4220Δ*Spa*, and BAA1717). Vaccinated mice had increased CD4+ and CD8+ T cells in the adjacent draining lymph node. Vaccination induced higher IgG1 and IgG2b whereas infection only increased IgG2b (Fig. 1A). Vaccinated animals had bioluminescent similar to uninfected animals by Day 45 (Fig. 1B). Vaccinated animals had significantly less implant bacteria than unvaccinated animals and 25% of vaccinated animals did not have any detectable bacteria at euthanasia (Fig. 1C).

**Figure 1.**
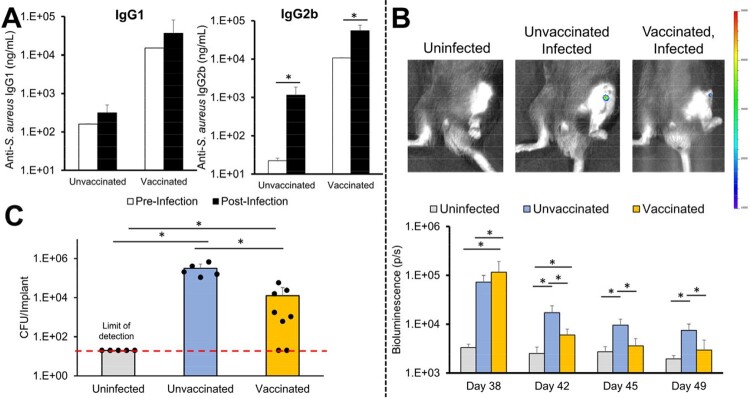
A) Anti-S. aureus immunoglobulins pre- (Day 28) and post-infection (Day 49). B) Representative bioluminescent images at Day 42 and quantification. C) Implant bacterial burden at euthanasia (Day 49). * = p<0.05.

**Conclusion:**

Scaffold-based vaccination increased generalized anti-*S. aureus* antibody production in an antigen dose-dependent manner and decreased bacterial burden in a murine model of PJI.

**Disclosures:**

**David Mooney, Ph.D.**, Advanced Healthcare Materials: Board Member|Agnovos: Stocks/Bonds|Attivare: Stocks/Bonds|Boston Scientific: Advisor/Consultant|Cartesian Therapeutics: Advisor/Consultant|IVIVA: Stocks/Bonds|Johnson & Johnson: Advisor/Consultant|Journal of Biomedical Materials Research: Board Member|Lightning Bio: Stocks/Bonds|Lyell: Stocks/Bonds|Medicenna: Advisor/Consultant|Norvartis: Advisor/Consultant|Norvartis: Grant/Research Support|Norvartis: IP Royalties|Revela: Stocks/Bonds|Samyang: Advisor/Consultant

